# An unusual cause of subarachnoid haemorrhage in a patient with newly diagnosed neurofibromatosis: a case report

**DOI:** 10.4076/1757-1626-2-8399

**Published:** 2009-08-11

**Authors:** Chisha Weerasinghe, Prem Jesudason, Daniel Peckham

**Affiliations:** Department of Respiratory Medicine, St James University HospitalBeckett Street, Leeds, LS9 7TFUK

## Abstract

**Introduction:**

In this report we discuss an unusual cause of subarachnoid haemorrhage in association with neurofibromatosis.

**Case presentation:**

A previously fit 55-year-old man developed sudden onset headache with loss of consciousness. He was comatose on admission with no focal neurological signs. Numerous neurofibromas and café-au-lait patches were noted, indicating neurofibromatosis type 1 which had not been previously diagnosed. Computer Tomography brain revealed a grade IV subarachnoid haemorrhage in association with numerous vascular lesions on cerebral angiography.

**Conclusion:**

A rare cause of subarachnoid haemorrhages was identified and is discussed in detail.

## Introduction

The neurofibromatoses are genetic disorders of the nervous system that primarily affect the development and growth of neural cell tissues. The neurofibromatoses are classified as neurofibromatosis type 1 (NF1) and neurofibromatosis type 2 (NF2). NF1 is the more common subtype and the gene responsible is located at the chromosome region 17q11.2 [1,2]. Diagnostic criteria have been set out by the National Institute of Health for the clinical diagnosis of NF1 [3] and a diagnosis can be made if two or more of the following features are present:

- two or more neurofibromas or one plexiform neurofibroma,
- six or more café-au-lait macules larger than 15 mm post-puberty (or larger than 5mm pre-puberty),
- Axillary/inguinal freckling (Crowe’s sign),
- Optic glioma,
- two or more iris hamartomas (Lisch nodules),
- A distinctive osseous lesion (for example long bone dysplasia),
- A first degree relative with NF1.

## Case presentation

**Figure 1.**
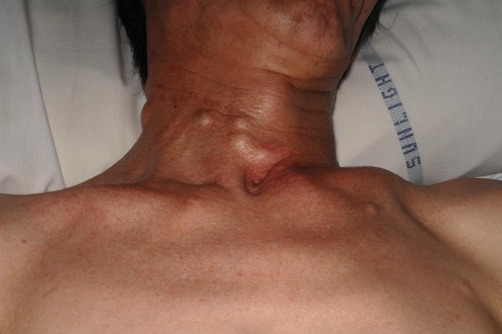
Neurofibromas; multiple neurofibromas were present on the patient’s neck, as shown. They were also present on his arms and abdomen.

**Figure 2.**
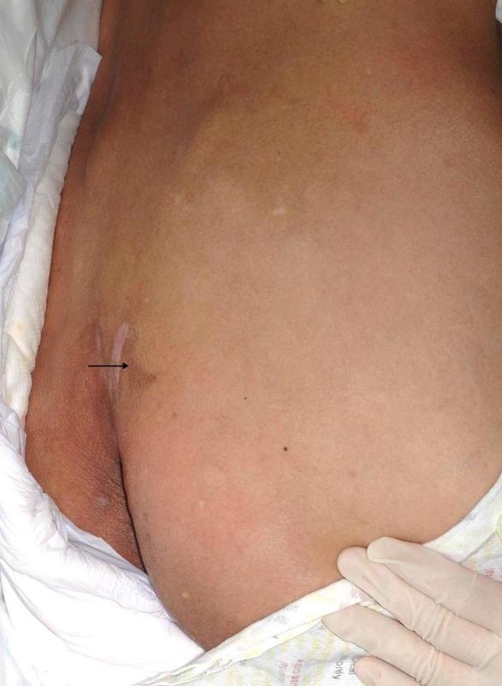
Café-au-lait patches; multiple café-au-lait patches were present on the patient’s skin. Shown here (arrow) is a café-au-lait patch on the patient’s lower back.

A 55-year-old male of Chinese origin in good health and on no regular medications developed an acute headache and subsequently lost consciousness. On admission he was comatose with a GCS of 5 with no localizing neurological signs. His blood pressure was 136/83mmHg and his vital signs were stable. At this time it was noted that his neck, arms and abdomen were covered with numerous, ‘lumps’ (Figure 1) and he also had multiple brown patches on his skin (Figure 2). The skin lumps were most likely neurofibromas and the patches were café-au-lait spots, consistent with a diagnosis of neurofibromatosis type 1. He had never sought medical advice for this and there was no previous family history of neurofibromatosis. Additionally, he had no past history or family history of hypertension or neurological disease.

**Figure 3.**
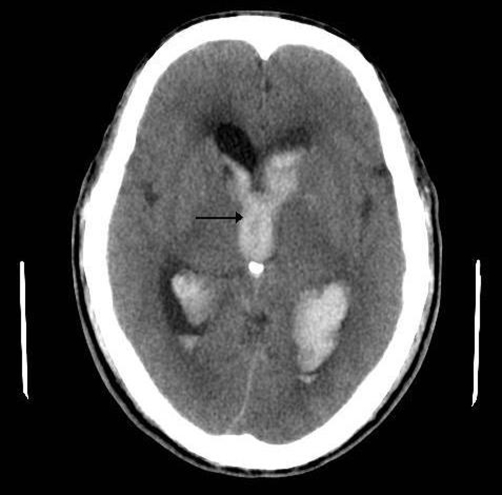
CT head; CT scan of the patient’s head demonstrated high attenuation areas in the ventricles, indicating intraventricular bleeding (arrow).

A CT head scan revealed a grade IV subarachnoid haemorrhage with intraventricular blood and dilated ventricles suggestive of hydrocephalus (Figure 3). Extraventricular drainage of cerebrospinal fluid was performed and the patient’s GCS improved but he did not regain full speech or mobility.

To further characterise the origin of the bleed and to identify any longstanding changes in cerebral vasculature, cerebral angiograms were performed. The angiogram revealed an underlying diagnosis of moyamoya disease: a disease of cerebral vasculature, and one that has a rare association with neurofibromatosis [1].

**Figure 4.**
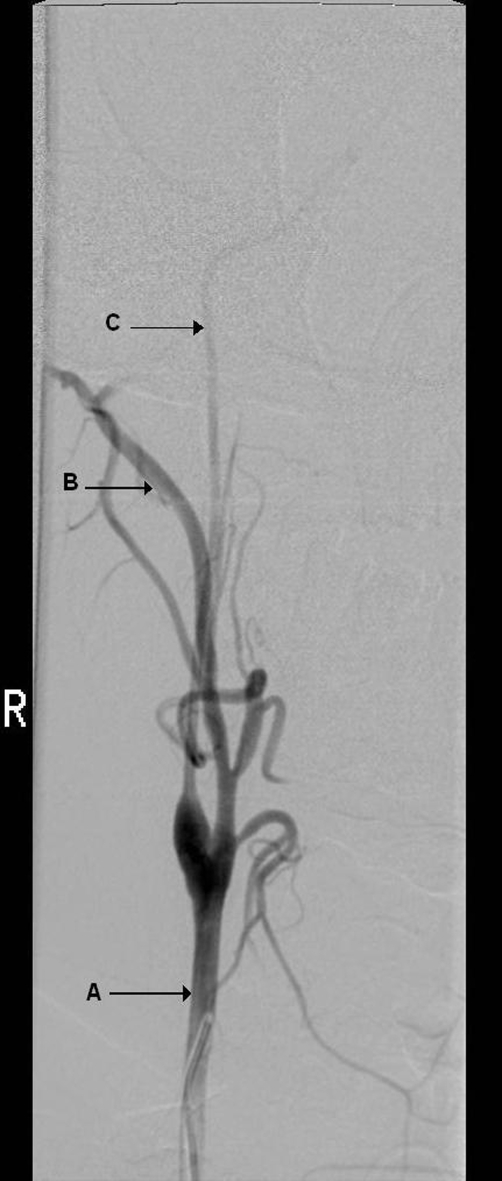
Angiogram demonstrating occlusion of right internal carotid artery; the common carotid artery is shown (arrow A) and distal to the carotid bifurcation the external carotid fills normally (arrow B). However, the internal carotid artery is occluded (arrow C). These changes were seen bilaterally.

**Figure 5.**
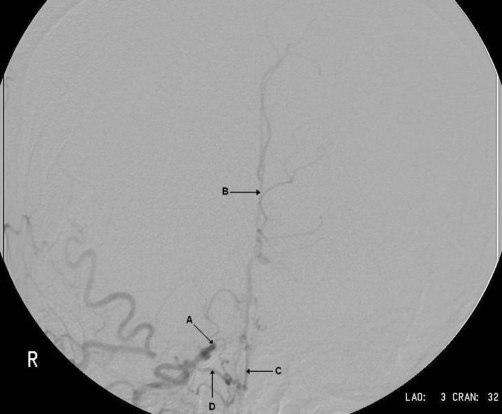
Angiogram demonstrating changes in anterior circulation; the occluded right internal carotid artery is seen (arrow A), this time in a coronal view. The anterior cerebral artery (arrow B) is being perfused, but not by the internal carotid artery. Instead the anterior circulation is dependent upon collateral vessels (arrow C), that originate from the ophthalmic artery (arrow D).

**Figure 6.**
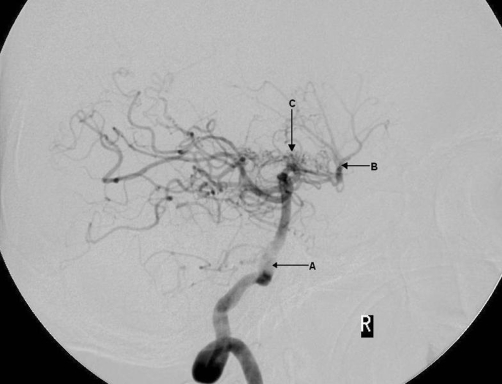
Angiogram demonstrating posterior circulation and moyamoya vessels; a sagittal view in which the basilar artery (arrow A) is seen filling the middle cerebral artery (arrow B). This is occurring due to the occlusion of the internal carotid arteries. Also shown are the moyamoya vessels (arrow C) in a ‘puff of smoke’ formation (see ‘discussion’). These vessels have developed secondary to occlusion in and around the circle of Willis.

Cerebral angiography demonstrated intracranial occlusion of the internal carotid arteries (Figure 4), a hallmark of moyamoya disease. Due to the stenosed internal carotid arteries, blood supply to the anterior cerebral arteries was dependent upon collateral vessels (Figure 5). Supply to the middle cerebral arteries originated from the basilar artery, and furthermore, occlusion in and around the circle of Willis had led to the formation of hypertrophied basal perforator vessels, known as moyamoya vessels (Figure 6). It was likely that the subarachnoid haemorrhage occurred secondary to rupture of a fragile perforator vessel. In summary, the angiograms demonstrated a diagnosis of moyamoya disease, which has a rare association with neurofibromatosis [1].

No further neurosurgical options were possible and the patient remains dependent for all care. He is undergoing neurosurgical rehabilitation in a specialized facility.

## Discussion

Moyamoya disease is a progressive occlusive disease of the cerebral vasculature with particular involvement of the circle of Willis and the arteries that feed it. It may present with progressive neurological symptoms including hemiparesis or sensory impairment, or as in this case with an acute event such as a subarachnoid haemorrhage. The name “moyamoya” comes from the Japanese for “puff of smoke” describing the characteristic angiographic appearance of the abnormal collateral arterial networks that develop adjacent to the stenotic vessels. The following findings on angiography in our patient support the diagnosis: 1) occlusion of the terminal portions of both internal carotid arteries and 2) abnormal vascular networks in the region of the occlusive areas [4].

It is a rare condition more commonly seen in the Japanese population where the prevalence is 3.16 cases per 100,000 people and the annual incidence is 0.35 per 100,000 people. With regard to sex, the female-to-male ratio is 1.8:1 [4]. The disease is believed to have a familial tendency with 10% of patients reporting a family history [5]. The gene responsible for familial moyamoya disease is located at chromosome 17q25 [1].

A rare association between NF1 and moyamoya disease has been described [1]. In a literature review twelve cases of NF1 and moyamoya disease with neurological presentations were described [1]. Five such cases presented with intraventricular haemorrhage similar to our patient [6-10]. One case presented with an ischaemic stroke. The remainder presented with progressive neurological symptoms and signs.

In general, patients with moyamoya disease that present with progressive symptoms are likely to have a better prognosis than those presenting with an acute event [4]. Many surgical interventions have been used in moyamoya disease, including superficial temporal artery-middle cerebral artery anastomosis, although the most effective technique is a matter of controversy [4].

NF1 associated with moyamoya disease presenting with intraventricular haemorrhage is a rare disease entity; however, considering the potential complications that can arise and that moyamoya disease may be familial, the diagnosis of NF1 in a family member could indicate a need for further screening. Further studies are needed to assess the benefits of early diagnosis and intervention in patients with moyamoya disease associated with NF1.

## Conclusion

We report a case of a previously undiagnosed patient with neurofibromatosis who developed a subarachnoid haemorrhage secondary to moyamoya disease. This unusual condition has classical radiological features and is one of many unusual complications of neurofibromatosis.

## Abbreviations

CT: Computer Tomography
GCS: Glasgow Coma Scale
NF: Neurofibromatosis

## References

[bib-001] Koc F, Yerdelen D, Koc Z (2008). Neurofibromatosis type 1 association with moyamoya disease. Int J Neurosci.

[bib-002] http://www.emedicine.com/radio/TOPIC474.HTM.

[bib-003] National Institutes of Health Consensus Development Conference (1988). Neurofibromatosis Conference statement. Arch Neurol.

[bib-004] http://www.emedicine.com/neuro/topic616.htm.

[bib-005] Fukui M (1997). Current state of study on moyamoya disease in Japan. Surg Neurol.

[bib-006] Khan M, Novakovic RL, Rosengart AJ (2006). Intraventricular hemorrhage disclosing neurofibromatosis 1 and moyamoya phenomena. Arch Neurol.

[bib-007] Siqueira Neto JI, Silva GS, De Castro JD, Santos AC (1998). Neurofibromatosis associated with moyamoya arteriopathy and fusiform aneurysm: case report. Neuropsiquiatr.

[bib-008] Fujimoto K, Shimomura T, Okumura Y (1999). Severe stenosis of the internal carotid artery and intracerebral hematoma associated with neurofibromatosis type 1: a case report. No Shinkei Geka.

[bib-009] Sasaki J, Miura S, Ohishi H, Kikuchi K (1995). Neurofibromatosis associated with multiple intracranial vascular lesions: stenosis of the internal carotid artery and peripheral aneurysm of the Heubner’s artery; report of a case. Shinkei Geka.

[bib-010] Erickson RP, Woolliscroft J, Allen RJ (1980). Familial occurrence of intracranial arterial occlusive disease (Moyamoya) in neurofibromatosis. Clin Genet.

